# Effectiveness of Chêneau brace treatment for idiopathic scoliosis: prospective study in 79 patients followed to skeletal maturity

**DOI:** 10.1186/1748-7161-6-2

**Published:** 2011-01-25

**Authors:** Katarzyna Zaborowska-Sapeta, Ireneusz M Kowalski, Tomasz Kotwicki, Halina Protasiewicz-Fałdowska, Wojciech Kiebzak

**Affiliations:** 1 Department of Rehabilitation, Faculty of Medical Sciences, University of Warmia and Mazury, Olsztyn, Poland; 2 Children Rehabilitation Hospital, Olsztynek, Poland; 3 Department of Pediatric Orthopedics and Traumatology, University of Medical Sciences, Poznan, Poland; 4 Institute for Physiotherapy, Faculty of Medical Sciences, University of Kielce, Poland

## Abstract

**Background:**

Progressive idiopathic scoliosis can negatively influence the development and functioning of 2-3% of adolescents, with health consequences and economic costs, placing the disease in the centre of interest of the developmental medicine. The aim of this study was to evaluate the effectiveness of Chêneau brace in the management of idiopathic scoliosis.

**Methods:**

A prospective observational study according to SOSORT and SRS recommendations comprised 79 patients (58 girls and 21 boys) with progressive idiopathic scoliosis, treated with Chêneau brace and physiotherapy, with initial Cobb angle between 20 and 45 degrees, no previous brace treatment, Risser 4 or more at the final evaluation and minimum one year follow-up after weaning the brace. Achieving 50° of Cobb angle was considered surgical recommendation.

**Results:**

At follow-up 20 patients (25.3%) improved, 18 patients (22.8%) were stable, 31 patients (39.2%) progressed below 50 degrees and 10 patients (12.7%) progressed beyond 50 degrees (2 of these 10 patients progressed beyond 60 degrees). Progression concerned the younger and less skeletally mature patients.

**Conclusion:**

Conservative treatment with Chêneau orthosis and physiotherapy was effective in halting scoliosis progression in 48.1% of patients. The results of this study suggest that bracing is effective in reducing the incidence of surgery in comparison with natural history.

## Background

Idiopathic scoliosis is a developmental deformation of the spine and the trunk, which significantly influences the form and function of a young organism. The extensive interest of medical experts in the treatment of spinal deformities results from the incidence of such disorders in the adolescent population (2-3%), health consequences of the disease progression as well as social and economic costs [1-4]. On the other hand, the health related quality of life of the adults with mild to moderate idiopathic scoliosis, including individuals conservatively treated in adolescence, seems very good [5-7]. Bracing and physiotherapy are the non-surgical methods of treatment practiced for mild to moderate scoliosis. The aim of brace treatment is to stop deterioration of the deformity, which is a natural history of progressive scoliosis beyond 25 degrees in immature adolescents. Within the group of rigid Thoraco-Lumbo-Sacral Orthoses (TLSO), the Cheneau brace is most widely used in Poland. The major mechanism of this orthosis consists of correcting three-dimensional deformity of the spine and the trunk by a system of multipoint pressure zones and expansion chambers [8,9]. Studies carried out so far have shown that wearing a brace changes the natural history of scoliosis and probably helps the patient to avoid surgical procedure [10-12], especially if the brace follows current quality standards [13]. One meta-analysis has shown that bracing is an effective therapeutic method for idiopathic scoliosis [14]. In 2005 a systematic review of literature was carried out focusing on evaluating the effectiveness of conservative treatment methods for scoliosis, including bracing [15]. Out of 436 articles only 3 discussed randomized studies and 10 included a control group. However, only 5 referred to bracing. A comparison of a brace treated group with a control group showed a significant superiority of bracing [16]. Another study on the effectiveness of bracing (Milwaukee) as a supplementary treatment for exercising did not show therapeutic effect; the results of both groups did not differ statistically [17]. A comparison of bracing with exercises did not show difference between the groups [18]. However, a comparison of bracing with electrostimulation showed a higher effectiveness of the former therapy [19]. A comparison of various braces (Charleston Bending Brace, Milwaukee) did not reveal a significant advantage of any of them [20-22]. Finally, recent Cochrane Database of Systematic Reviews publication revealed a low quality evidence in favour of using bracing [23].

The aim of this study was to assess the effectiveness of the Chêneau brace [24] in a series of patients having achieved skeletal maturity.

## Methods

Study design: prospective observational study, relying on the SOSORT [13] and SRS [25] criteria for brace studies.

302 new patients were found in the database while 192 of them were actively treated for progressive scoliosis in our institution between 2003 and 2008. Among the 302 patients, 54 of them were seen once and received the recommendation for conservative scoliosis treatment but never came back to start the treatment. Further 56 patients initiated the conservative treatment in our institution by receiving the orthosis and initial exercise training; they subsequently continued the treatment elsewhere, near their place of residence for convenience reasons; those 56 patients stayed inaccessible for us for the final evaluation. Thus, the overall number of patients actively treated in our institution was 192. All 192 patients were prospectively evaluated using the computer database. The inclusion criteria for this study were as follows: both sexes, diagnosis of idiopathic scoliosis, initial Cobb angle between 20 and 45 degrees, no previous brace treatment, Risser 4 or more at the final evaluation, minimum one year follow-up after weaning the brace. All conservatively treated patients with scoliosis of 20° to 29° had a radiological evidence of progression (defined as Cobb angle increase of 5° or more in 6-month interval). The patients with scoliosis of 30° or more started the brace treatment without a radiological evidence of progression.

Standing out-of-brace frontal Cobb angle [26] was measured before the treatment and at the follow-up. The vertebral axial rotation was quantified at the level of the apical vertebra according to the Cobb method [27]. The Risser sign was assessed according to the United States description [28]. The single (thoracic, thoracolumbar or lumbar) and the double curves (thoracic and lumbar or thoracic and thoracolumbar) were distinguished.

**Figure 1:**
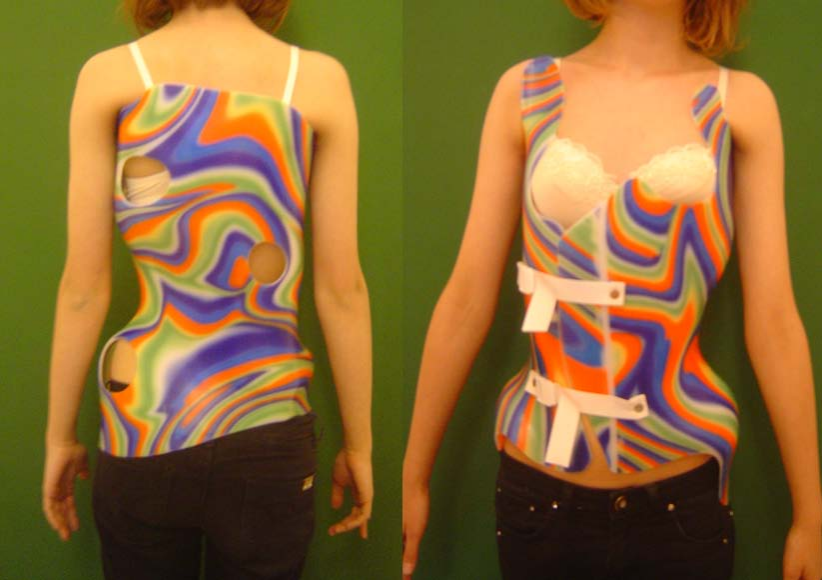
**Chêneau brace conceived for double idiopathic scoliosis: right thoracic and left lumbar**. Left: back view. Right: front view.

The patients received the brace therapy (Figure 1) according to the Chêneau principles [24] for 20 hours per day, together with physiotherapy comprising active asymmetric exercises based on proprioceptive neuromuscular facilitation technique. The treatment was initiated through a 21-day in-patient rehabilitation stay that was dedicated to learning physiotherapy and fitting the brace. Subsequent out-patient management consisted of regular clinical examination and control of the brace and exercises at 3-month intervals. Radiographic control was made once a year with AP standing out of brace radiograph, then one year after weaning the brace. The weaning of the brace was started at Risser 4 and 2-year-postmenarche; it was carried on gradually, first for school hours, then for the rest of the day, finally for the night hours.

The outcome was assessed based on the SRS criteria. According to the Cobb angle the patients were classified as: (1) **improved**: decrease of the Cobb angle by 6 degrees or more, (2) **stable**: no more than 5 degrees of progression or improvement, (3) **progressed**, with the Cobb angle less than 50 degrees, and (4) **progressed **beyond the Cobb angle of 50 degrees who were considered candidates for surgery. Additional analyses of the age, angle and treatment duration were performed within each subgroup.

## Results

Seventy-nine out of 192 patients met the inclusion criteria. The excluded patients included: (1) skeletally immature and still under treatment - 66 patients and (2) non-idiopathic curvatures - 47 patients. Among the 79 patients there were 58 girls (73.4%) and 21 boys (26.6%).

At follow-up 20 patients (25.3%) improved, 18 patients (22.8%) were stable, 31 patients (39.2%) progressed below 50 degrees and 10 patients (12.7%) progressed beyond 50 degrees (2 of these 10 patients progressed beyond 60 degrees). The detailed data is presented in Table 1.

**Table 1:** Patients data according to the four outcome subgroups

	Improved	Stable	Progressed below 50°	Progressed beyond 50°	Total
Number of patients	20	18	31	10	79

%	25.3	22.8	39.2	12.7	100.0

Single/Double	7/13	7/11	20/11	5/5	39/40

Initial age (years)	14.3 12-16	13.8 13-17	13.7 13-15	12.4 10-15	13.5 10-17

Initial Cobb angle (°)	33.5 23.0-45.0	30.8 22.0-38.0	31.2 22.0-39.0	34.3 27.0-45.0	32.9 22.0-45.0

Initial rotation (median)	3	2	3	3	3

Initial Risser (median)	3	2	0	0	1

Bracing time (years)	2.6 2.0-5.0	2.4 1.0-4.0	3.7 2.0-5.0	2.3 1.0-4.0	2.7 1.0-5.0

Follow-up Cobb angle (°)	23.2 12.0-39.0	32.3 26.0-40.0	39.0 33.0-46.0	57.2 53.0-72.0	37.9 12.0-72.0

Follow-up Cobb change (°)	-9.8	± 5.0	+7.7	+15.0	+9.2

Follow-up rotation (median)	3	2	3	3	3

N - number of patients; Single/Double- number of patients with Single versus Double curvatures.

Mean value and the range are given for age, Cobb angle and duration of bracing.

## Discussion

Our results confirm the findings of other researchers that the management of progressive idiopathic scoliosis with corrective bracing and physiotherapy alters the natural history of the disease. We were able to stop the progression in 48.1% of patients and to slow down the rate of progression in additional 39.2%. The percentage of patients in whom the Cobb angle exceeded 50 degrees (12.7%) and therefore had surgical recommendation may be considered comparable to the literature data. On the other hand, we were not fully satisfied with a relatively large percentage of patients in whom the intended therapeutic effect of stopping progression was not achieved (51.9%). Progression was observed mainly in the youngest and least skeletally matured children (Table 1.). The authors recognize that they have not followed the recommendation to consider the progression as the 10°of Cobb angle difference for the curves under 25°. However, there were only three patients with the curves of 20 to 24° (two of them had a curve of 22° and one had a curve of 23°of Cobb). Also, we cannot be sure that all patients being over 30° at the treatment initiation represented progressive scoliosis.

In the 1995 Nachemson's et al. study on wearing a brace demonstrated better outcome than observation alone [16]. However, the research carried out by Goldberg et al. on the effectiveness of the Milwaukee brace and the TLSO showed that bracing did not influence the rate of surgical procedures. In the control group, the surgical procedures amounted to 28.1%, while in the braced group to 24%, which was not significantly different [29]. Further publications demonstrated a lower rate of surgery in conservatively treated patients. Maruyama et al. studied a large group of 328 patients. In 20 patients (6.1%), scoliosis progression exceeding 50° was observed and those patients were qualified for surgery. The age of initiating the bracing in this subgroup was 13.4 years, the Cobb angle was 48.5°, the mean age of surgery was 16.0 years, whereas the angle of curvature after the bracing was 62.2° [30]. Rigo et al. studied a group of 157 children qualified for bracing. During the observation period, 13 patients did not complete the therapy, whereas 6 were qualified for surgical treatment with the mean Cobb angle of 61.5°. Thus, the frequency of surgery was 3.8%. Assuming that the patients who did not complete the therapy would also undergo surgical treatment, the surgery rate would be of 12.1% [11]. Negrini et al. conducted a prospective study of 112 patients; the complete data of 108 patients, aged 13.2 ± 1.8 years, with initial Cobb angle 23.4 ± 11.5° was available. One person underwent corrective spinal procedure, which makes the frequency of surgery at the level of 0.9%, and assuming that the patients who did not complete the treatment would also undergo surgery - at the level of 4.5% [31]. Weiss et al. in a retrospective study of the patients treated with the Chêneau brace between 1993 and 1996 in Bad Sobernheim, analyzing 343 girls, having the angle of curvature of 33.4° found 41 patients having underwent operation, which makes the rate of surgery 11.9% [12]. Lange et al. in a retrospective study reported good long-term results of Boston brace treatment with the rate of 6.5% of patients who underwent scoliosis surgery [32].

In this study, the number of patients that we initially debated to consider as the drop-outs seemed very high. In fact, in our opinion these patients do not fulfill the criteria for being considered the real drop-outs. They may be considered as the patients who were not treated in our institution. We disclosed their number for clarity, however we cannot feel responsible for their course of the disease. The issue concerns the organization and logistics of the health care services in our country, and the patients' attitude. Thus, it is not be used to assess the quality of conservative scoliosis treatment. We were aware that the parents of 56 patients continued managing their child at proximity of the place they lived, however we are not able to assess the quality and the outcome of such a management. Moreover, we had no data of the 54 patients who received brace recommendation but never appeared after the first visit. We suppose that some of them did not accept this form of therapy while some found the orthosis providers elsewhere. Unfortunately, we are afraid of the quality of the orthoses because we could observe examples of various constructions, often erroneous, of plastic devices delivered under the name of Chêneau by negligent producers. This made us express our opinion on the need for registering new patients receiving conservative scoliosis treatment and for the standardization of the orthotic treatment in our country.

We paid extreme importance to psychological support in order to decrease the stress and increase the compliance. We noticed that Chêneau brace was unwillingly accepted by adolescents because of aesthetic and functional reasons. The brace was considered, especially by girls, as an element making every day life activities difficult. In our opinion, the role of regular physiotherapy specifically adjusted to the type and degree of scoliosis is crucial. The lack of determination to wear the brace and to follow physician's recommendations was a regularly observed risk. The patients undergoing the brace treatment having scoliosis at the angle of 40° or more were referred for an orthopedic consultation in order to consider the plan of surgical intervention. Afterwards, we observed that the motivation of these patients and the family to continue the conservative treatment strengthened in most cases.

## Conclusion

Conservative treatment with Chêneau orthosis and physiotherapy was effective in stopping scoliosis progression in 48.1% of patients. The results of this study suggest that bracing is effective in reducing the incidence of surgery in comparison with natural history.

## Competing interests

The authors declare that they have no competing interests.

## Authors' contributions

KZS and IMK conceived the study, collected data and drafted the manuscript, TK participated in the design of the study, interpreted the data and drafted the manuscript, HPF and WK collected data and helped to draft the manuscript. All authors read and approved the final manuscript.
